# A global dataset of native and alien distributions of alien species

**DOI:** 10.1038/s41597-025-06379-6

**Published:** 2025-12-04

**Authors:** Manuela Gómez-Suárez, Philipp Laeseke, Hanno Seebens

**Affiliations:** Ecological Informatics, Department of Animal Ecology & Systematics, Justus Liebig University, Giessen, Germany

**Keywords:** Invasive species, Biogeography

## Abstract

Biological invasions have been identified as a major threat to nature with far-reaching consequences also for human-wellbeing. Mitigating and preventing further impacts requires knowledge about the origins of alien species and their spread dynamics. While several new datasets have been published recently about alien species distributions, information about their native ranges is scarce and scattered. Here, we present a comprehensive dataset of the regions of origin and introduction of alien species worldwide. We accessed multiple global datasets of species distributions to compile, harmonize and integrate data of alien species distributions and their native ranges. Building on previous efforts, we advanced an existing workflow to allow full reproducibility and transparency in creating the dataset. The final dataset contains 427,956 records including alien distributions of 39,700 species and native distributions of 21,345 alien species in 289 regions worldwide. This dataset provides a solid foundation for analyses of alien species flows, the identification of new emerging alien species and the management of biological invasions through prioritizing major routes of spread.

## Background & Summary

The human-mediated introduction of species outside their native range - a process called biological invasions - can lead to alterations in biological communities and ecosystems, impose economic costs and negatively affect human well-being^1^. Indeed, biological invasions have been identified as a major driver of biodiversity loss^2^. This driver has intensified continuously over decades and is expected to rise further in the future^3,4^. Due to the urgency of addressing this issue, global biodiversity and sustainable development targets, such as target 6 of the Kunming Montreal Global Biodiversity Framework^5^ and target 15.8 of the Sustainable Development Goals^6^ call for the implementation of measures to prevent the introduction of alien species. To support these efforts, comprehensive information on the distributions of species both in their native and introduced ranges is key to assess progress towards these goals^7^, and to pinpoint major source regions of alien species, hotspots of introduction and vulnerable introduction areas^8^.

In order to address the need for distributional data of alien species, several studies and datasets have been published mapping the regions of introduction (i.e. alien distributions) of alien species^4,9–15^. However, limited efforts have been made to compile information on the regions to which these alien species are native (i.e. native distributions). When such records are available^10,16^, they encompass a small subset of species and are often compiled at a broad geographic scale, such as continents or realms. Understanding the native distributions of these species is equally critical, as they provide insights into hotspots of alien species’ origins, as well as of the species’ ecological niches. This information is required to identify new emerging alien species through horizon scans or predictive modelling, thus facilitating the development of mitigation strategies^17,18^.

Prior initiatives have made significant contributions regarding the standardization and compilation of alien species distributional data and information regarding these introductions. The Standardizing and Integrating Alien Species (SInAS) workflow^19^ focuses on standardizing and integrating information for alien species distributions, including characteristics related to the alien status of a species in a location (e.g. year of first introduction record and degree of establishment, among others). Additionally, several datasets, such as a Spatio-temporal Dataset of Global Invasive and Alien Species and their Traits (GIATAR)^10^, the Country Compendium of the Global Register of Introduced and Invasive Species (GRIIS)^9^ and the First Records dataset^4^, have been compiled by integrating datasets on species distributions across introduced regions. Despite these efforts, a critical gap persists in understanding comprehensive biogeographic histories, as these datasets primarily document the presence of species in alien regions, while native distributions are usually missing.

To address this gap, we extended the SInAS workflow and compiled new data to build the SInAS dataset 3.1.1^20^ (10.5281/zenodo.5562891), a compendium of alien and native distributions of alien taxa based on published checklists (i.e. regional species lists) and expert-based range maps. This dataset provides information for 289 locations, representing non-overlapping administrative units of the terrestrial Earth. It covers a wide range of alien taxa, including plants, animals, fungi and microorganisms. It contains 427,956 records, including alien distributions for 39,700 alien taxa and native distributions for 21,345 alien taxa (see *Data overview* for further details).

## Methods

Our overall approach is divided into three steps: Defining the structure of the SInAS dataset, data compilation and data harmonization. Data compilation consisted of screening available data sources of native and alien species distributions and gathering available information regarding their distribution. Data harmonization comprised the standardization and integration of the compiled data sources according to available standards in biodiversity research. All steps were conducted in R (v 4.4.2)^21^. The compilation, harmonization and visualization of the data was done using the packages data.table (v 1.16.2)^22^ and tidyverse (v 2.0.0)^23^.

### SInAS dataset structure

As final data products, two datasets were produced: A table containing distributional information of species and a second table containing taxonomic information of the species.

The first table provides information about the taxon, location, invasion status (means of establishment, degree of establishment, occurrence status and pathway), year of first record and habitat (Table 1). Each row corresponds to a record of a taxon in a given location being either *alien* or *native*. Thus, a species could potentially have an entry of *alien* and *native* occurrence in the same region. We followed as closely as possible the existing standard in biodiversity research called Darwin Core terminology^24^, as well as recent proposed standards for harmonizing data on the invasion status of a species^25^. These resources provide glossaries of terms that facilitate the sharing of biodiversity information. Accordingly, invasion status is separated into the columns *occurrenceStatus*, *establishmentMeans* and *degreeOfEstablishment*. In some cases, we incorporated new columns considering information relevant for our case, which are not reflected in Darwin Core (e.g., *locationID* to provide a unique ID for each location).

**Table 1:** Fields and field terms in the SInAS dataset.

Field	Data type	Description	Terms in field
location	character	Location name where a species is present	e.g. Colombia
locationID	integer	Unique internal identifier of a location	e.g. 58
taxon	character	Taxon name without authority and date information	e.g. *Lonchura malacca*
taxonID	integer	Unique internal identifier of a taxon	e.g. 9697
eventDate	integer	Year of first record of introduction of a taxon in a location	e.g. 2010
habitat	character	Variable indicating the habitat type where a taxon is found. Multiple habitats can be assigned to a taxon.	brackish; freshwater; marine; terrestrial
occurrenceStatus	character	Variable indicating the presence or absence of a taxon in a location. A value of *absent* refers to a species that was once recorded in a location, and is now absent (e.g. considered to be locally extinct or eradicated from that location).	present; absent
establishmentMeans	character	Variable indicating whether a taxon is native or has been introduced to a given location	native; introduced; vagrant; uncertain
degreeOfEstablishment	character	Variable indicating how a taxon survives, reproduces and expands its range in a location	established; reproducing; invasive
pathway	character	The process by which a taxon came into a given place at a given time following the classification by CBD^54^	e.g. escape from confinement; release in nature
datasetName	character	Information regarding the original source or dataset of a given record	e.g. GAVIA
bibliographicCitation	character	Full reference to the original source (datasetName)	e.g. Dyer, E., Redding, D. & Blackburn, T. Data from: The Global Avian Invasions Atlas - A database of alien bird distributions worldwide. Figshare 10.6084/M9.FIGSHARE.4234850.V1 (2016)

We included an example entry for the fields with no fixed terms.

The second table provides taxonomic information for each taxon, including the taxonomic name as provided in the original data source and higher taxonomic information as retrieved from the Global Biodiversity Information Facility (GBIF) Backbone Taxonomy^26^ (Table 2). Note that we use both terms, taxon and species, in the text for readability but refer only to taxon in the dataset as it contains also subspecies, hybrids and variants.

**Table 2:** Fields and field terms in the taxonomic tree file included with the SInAS dataset.

Field	Data type	Description	Terms in field
scientificName	character	Full scientific name of a taxon; with authority and date information if known	e.g. *Lonchura malacca* (Linnaeus, 1766)
verbatimTaxonRank	character	Original taxon name as provided by the original dataset	e.g. *Lonchura malacca*
taxon	character	Taxon name without authority and date information	e.g. *Lonchura malacca*
GBIFstatus	character	Status of the use of the scientificName according to GBIF	ACCEPTED; SYNONYM; MISSING
GBIFstatus_Synonym	character	Variable indicating whether a scientificName was matched as a synonym with GBIF Backbone Taxonomy	ACCEPTED; SYNONYM; DOUBTFUL
GBIFmatchtype	character	How the taxon name was matched with GBIF	EXACT; FUZZY
GBIFtaxonRank	character	Taxonomic rank of the most specific name in the scientificName	SPECIES; GENUS; SUBSPECIES; FORM; VARIETY; FAMILY
GBIFusageKey	integer	Unique identifier for the name usage as documented in GBIF Backbone Taxonomy of the currently valid (zoological) or accepted (botanical) taxon	e.g. 2493626
GBIFnote	character	Further information on the taxon regarding taxon matching with GBIF	Homonym in GBIF; Multiple accepted names for synonym in GBIF; Multiple synonyms in GBIF; Accepted name found on GBIF
species	character	Name of the species epithet of the scientificName	e.g. *Lonchura malacca*
genus	character	Full scientific name of the genus in which a taxon is classified	e.g. *Lonchura*
family	character	Full scientific name of the family in which a taxon is classified	e.g. Estrildidae
order	character	Full scientific name of the order in which a taxon is classified	e.g. Passeriformes
class	character	Full scientific name of the class in which a taxon is classified	e.g. Aves
phylum	character	Full scientific name of the phylum or division in which a taxon is classified	e.g. Chordata
kingdom	character	Full scientific name of the kingdom in which a taxon is classified	e.g. Animalia
taxonID	integer	Unique internal identifier of a taxon	e.g. 9697
taxaGroup	character	Variable indicating the common name of the taxon group assigned to a taxon if any	Amphibians; Birds; Mammals; Reptiles; Vascular plants; Fishes; Insects; Arachnids; Crustaceans; Other arthropods; Molluscs; Fungi; Annelids; nematodes, platyhelminthes and other worms; Other aquatic animals; Algae; Bryophytes; SAR; Bacteria and protozoans; Viruses.

We included an example (e.g.) entry for the fields with no fixed terms.

For further information on the harmonization and final format of the fields see *Data harmonization* below.

### Spatial delineation

Records for alien taxa were compiled at the level of countries, islands or other sub-national geographic units, hereafter referred to as locations. While the main geographic unit in our dataset represents administrative boundaries of countries, we kept information for some well-studied sub-national units. These geographic units were selected because of their large (bio-)geographic distance to the mainland (e.g. Hawaii, Galapagos Islands or Shetland Islands), or because there is a significant amount of research and information on invasion biology specifically for the respective geographic unit (e.g. Alaska, Vancouver Island or Tasmania). Small islands close to the mainland of a country were not considered separately. The geographic boundaries of each geographic unit were retrieved from the Global Administrative Areas dataset (GADM; https://gadm.org/). The national divisions correspond to GADM’s level 0 boundaries, while the sub-national divisions correspond to level 1 or level 2 boundaries.

**Fig. 1:**
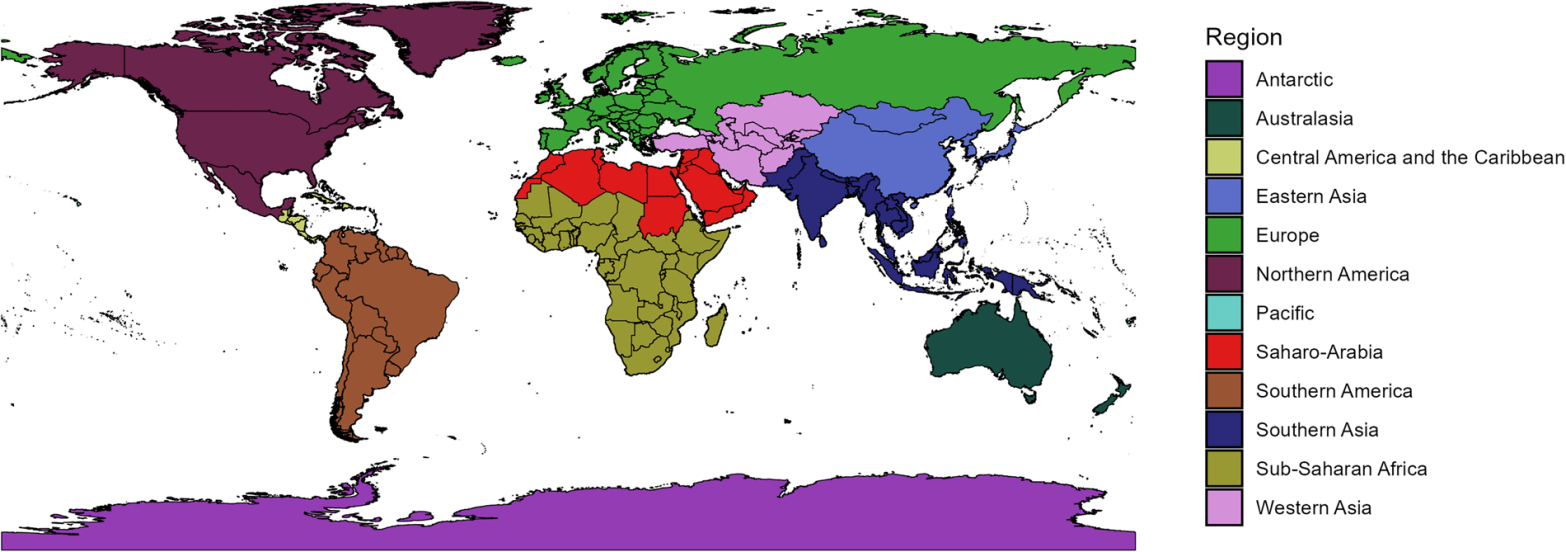
The 289 individual locations present in the dataset, as well as the custom regional aggregation of the locations.

**Fig. 2:**
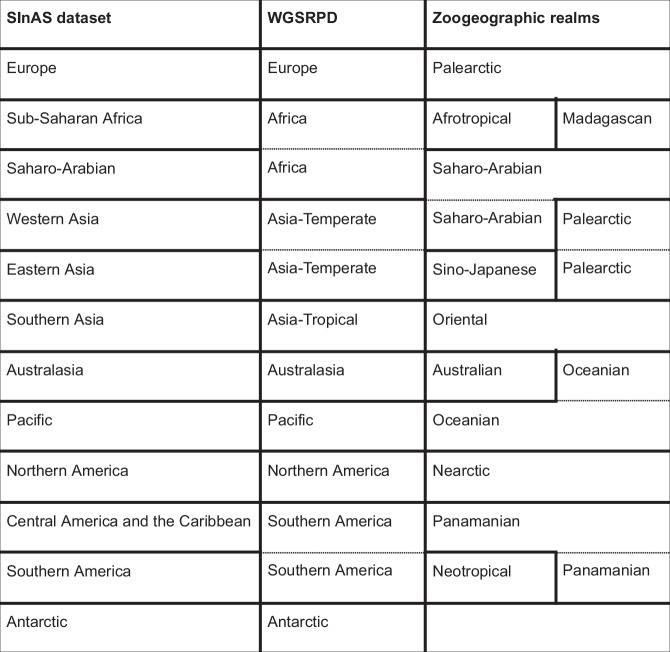
Overview on the regional aggregation used for this study (SInAS, Fig. 1), as well as the corresponding regions according to different biogeographical standards, such as the World Geographical Scheme for Recording Plant Distributions (WGSRPD) and the Zoogeographic realms described by Holt. Thicker and dotted lines accentuate the different grouping of the regions according to each standard. The countries and islands assigned to each region are visualized in Fig. 1.

The dataset contains a total of 289 non-overlapping locations grouped into a custom regional aggregation (Fig. 1). To obtain a feasible and useful set of regions, we oriented our categorization of regions along available and widely used delineations in biodiversity research, such as the World Geographical Scheme for Recording Plant Distributions^27^ (WGSRPD, also called TDWG regions) and the zoogeographic realms defined by Holt *et al*.^28^ (Fig. 2). In a few cases, we deviated from existing categorizations and separated larger regions to reflect known dynamics of invasion hotspots and spreading^29^. For instance, we sub-divided regions like Africa, Asia-Temperate and Southern America of WGSRPD; and the Palearctic, Oceanian and Panamanian zoogeographical realms as indicated in Fig. 2. Our geographical reference system allows the user to group the results according to individual needs following other biogeographical standards or a custom set of regions.

### Data compilation

As input for the data harmonization process, we searched for relevant datasets of alien species distributions and their native ranges. These datasets should provide records which could be transformed into our target format (see *SInAS dataset structure*). We restricted our search to datasets by the following criteria:

1. Provide information at the species or higher taxonomic level.
2. Provide distributional information either as checklists (i.e., species lists for a given area) or range maps.
3. Provide information on the means of establishment of an alien species (e.g. native or introduced) in a reported region.
4. Provide information at the global level.

We focused on global datasets as a first step to obtain good spatial coverage and comprehensive data. The integration of the many available regional inventories is beyond the scope and capacity of this study. However, such data is partly already integrated in the accessed datasets, and additional data can be added in subsequent steps.

We found 14 publicly available datasets and peer-reviewed scientific publications providing relevant information on alien species distributions and their native ranges (Table 3). Often, similar information is provided in multiple datasets of varying degree of comprehensiveness and quality. To avoid mixing individual records of different methodology and quality, we excluded some data: We excluded general datasets providing information of alien and native species distributions, if such information is provided in other specialized datasets. For example, the range maps by IUCN^30^ provide information about native and alien ranges for mammals. But spatial information of alien mammals is also provided by DAMA^12^, which is a new comprehensive dataset dedicated to alien mammal distributions. We therefore extracted the alien distributions of mammals only from DAMA and their native distributions from IUCN. Similar cases existed for vascular plants, birds, reptiles and amphibians. If such specific information was not available, we kept the records from the general datasets.

**Table 3:** Overview of the datasets used in this study, including the retrieved version (v), as well as the type of information extracted from each dataset.

Dataset name	Taxonomic groups	Native data	Alien data
FirstRecords, v 3.1^4,36^	Cross-taxonomic		x
GRIIS^9,37^	Cross-taxonomic		x
DAMA^12,34^	Mammals		x
GloNAF, v 3^13,31^	Vascular plants		x
GAVIA^11,33^	Birds		x
AmphRep^14^	Amphibians, reptiles		x
MacroFungi^15,32^	Fungi		x
GIATAR, v 3^10,38^	Cross-taxonomic	x	x
FishBase^41^	Fishes	x	x
WRiMS^45^ / WoRMS^43^	Cross-taxonomic (marine species)	x	x
Ants^35,50^	Ants	x	x
BirdLife, v 2023.1^53^	Birds	x	
IUCN, v 2024-2^30^	Amphibian, Reptiles, Mammals, Arthropods	x	
WCVP, v 13^49^	Vascular plants	x	

Where no specific dataset version is indicated, the version available at the time of publication was used. We assigned a name to datasets with no known acronym, e.g. for the datasets on amphibians and reptiles (AmphRep), fungi (MacroFungi) and Ants.

From each dataset, we extracted distributional and taxonomic information. If provided, we kept additional data regarding the preferred habitat of a taxon, as well as information about the invasion status of a taxon in a given location (Table 1).

### Alien distributions of alien species

Data on alien species and their alien distributions were obtained from eleven global datasets of alien species occurrences (Table 3): Six datasets are dedicated to alien distributions of a single taxonomic group each. Three are structured very similarly and provide checklists of alien species for multiple regions: one each on vascular plants (GloNAF^31^), macro fungi (MacroFungi^32^), and amphibians and reptiles (AmphRept^14^). Notably, two datasets, one each on birds (GAVIA^33^) and mammals (DAMA^34^) provide information both as checklists and range maps. In these cases, we accessed the checklists directly. We kept all records provided by these five datasets. For the sixth dataset, focused on alien ants^35^, only records regarding alien distributions flagged as *established outdoors* were kept. Note that, to our knowledge, this is the only taxon-dedicated dataset on the global distribution of alien insects.

Three datasets are cross-taxonomic, potentially including records from any taxonomic group: one with a focus on temporal dynamics of alien species (FirstRecords^36^), one on invasive species - alien species with negative environmental or socio-economic impacts - (GRIIS^37^) and one on alien species and their traits (GIATAR^38^). All records compiled by FirstRecords and GRIIS were kept.

The workflow behind the GIATAR dataset compiles data from four sources: the European and Mediterranean Plant Protection Organization Global dataset (EPPO-GD), CABI’s Invasive Species Compendium (CABI^39^), Delivering Alien Invasive Species Inventories for Europe (DAISIE^40^) and the Standardizing and Integrating Alien Species dataset (SInAS). We kept records originating from EPPO-GD and CABI, as both offer up-to-date information on the global distribution of alien species. Records from DAISIE on the distribution of alien species were not kept, as this dataset was last updated in 2005 and is not maintained anymore. Finally, in order to avoid duplicates, records from the SInAS dataset were not used in the harmonization process, as we already included the underlying datasets (GAVIA, GloNAF, DAMA, MacroFungi, AmphiRep, FirstRecords and GRIIS).

Lastly, two datasets focus on aquatic species, one for fishes (FishBase^41^), retrieved using the R package rFishBase^42^, and one for marine species (World Register of Marine Species, WoRMS^43^), retrieved using the R package worrms^44^. As FishBase is not only restricted to alien or invasive species, we only extracted the records flagged as *introduced* or *established*. For WoRMS, we restricted the search to the marine species listed as *alien* by the World Register of introduced Marine Species (WRiMS^45^). WRiMS is a specialized subset of WoRMS, specifically cataloguing the traits and distributions of marine species that have been introduced beyond their native ranges.

### Native distributions of alien species

We restricted our search on native distributions to those species with available alien distributions. Data on native distributions of alien species were obtained from seven global datasets of species occurrences or range maps (Table 3).

Two datasets with checklists have a taxonomic focus, one each on vascular plants (World Compendium of Vascular Plants, WCVP, https://sftp.kew.org/pub/data-repositories/WCVP/) and ants^35^. For plants, we kept the records labeled with a 0 for *introduced* and excluding those flagged with *location_doubtful*. For ants we kept those flagged as *native*.

Two datasets were included that provide range maps and species status within these ranges: one for birds (BirdLife International, https://datazone.birdlife.org/contact-us/request-our-data) and one for mammals, reptiles and amphibians (International Union for Conservation of Nature, IUCN, https://www.iucnredlist.org/resources/spatial-data-download). We kept only records flagged as *native* or *reintroduced*.

Finally, we accessed three cross-taxonomic datasets: From GIATAR, we kept all records flagged as *native*. The records regarding native distributions within GIATAR were compiled from a literature review by GIATAR’ authors, as well as from CABI, DAISIE and Takeuchi *et al*.^46^. Even though DAISIE records on the alien distribution were not kept due to the lack of maintenance of the dataset, we kept the records reporting the native distributions of alien species as these should still be valid. For fishes (FishBase) we kept the records flagged as *native* and *endemic*; and for marine species (WoRMS), we kept those flagged as *native*, *native - non endemic* and *native - endemic*.

### Data harmonization

Data harmonization involved two substeps, namely data preparation and data standardization. First, we checked the input data for the availability and format of the required variables and preprocessed them as needed (section *Data preparation)*. Afterwards, this information was standardized according to available standards in biodiversity research and in line with existing datasets. The standardized information was then integrated and cleaned to obtain the final dataset of native and alien distributions of alien species (section *Data standardization*).

These steps are based on the existing SInAS workflow, which we extended to incorporate native distributions.

### Data preparation

The selected source datasets provide information in different formats, which needed to be harmonized before applying the actual workflow.

Our workflow can handle checklists of species, while some datasets (IUCN and BirdLife) provide distributional data as range maps. Thus, we converted the range maps from IUCN and BirdLife reporting on the native distribution of alien mammals, amphibians, reptiles and birds into checklists. These range maps are provided as polygons in shapefiles. To extract the native distributions, we overlaid these polygons to our custom spatial layer of 289 non-overlapping locations (see *Spatial delineation*, Fig. 1). Afterwards, we extracted the names of the locations in the range maps using the function st_join of the R package sf (v 1.0-19)^47,48^.

Other aspects of datasets needed to be streamlined individually to facilitate the harmonization process. For example, where datasets such as WCVP and GIATAR provided information on a taxon’s means of establishment as binary variables (e.g. *0* for *introduced* and *1* for *native*), we converted this data into *native* or *introduced*. Finally, GloNAF and GAVIA provide information on the distributions of species across different columns, which we manually combined to the finest spatial level to obtain one single column of location records.

### Data standardization

Once the input datasets were brought into similar formats, we conducted the actual harmonization of records following the extended SInAS workflow. As an initial step, the availability of the variables required for running the workflow was checked. In our case, the required variables were the *taxon* name, the *location* where a species is present, as well as its means of establishment (*establishmentMeans*, e.g. *native* or *introduced*). Note that *establishmentMeans* is only required if the input dataset provides information of both native and alien occurrences and if the scope (native or alien) is filled for the full dataset (see *Usage notes* and manual for more details). Optional variables were also included, such as the invasion status (i.e. *occurrenceStatus*, *degreeOfEstablishment)*, the pathway of introduction *(pathway), and* the first reported introduction date (*eventDate*). The names of these columns were harmonized to facilitate the standardization in the next steps.

Next, the entries from these columns were standardized (Table 1). With the exception of taxon names and introduction dates, all variables were standardized using translation tables (provided along with this dataset, see *Usage notes*). These tables provide the final standardized terms along with their possible variations to match the original terms. Each standardization step returned the standardized terms, together with an additional file of matches and mis-matches to allow checking how and whether each record was translated.

Characteristics related to the status of each alien species in a given location (i.e. *occurrenceStatus, establishmentMeans*,*degreeOfEstablishment* and *pathway)* were standardized following Darwin Core’s standardization guidelines and terms^24,25^. Note that information on the degree of establishment and pathway only applies to the alien distribution of the species and thus is only available for such records. Additionally, introduction pathways are seldom detailed in original sources, and when such data is provided, its standardization is challenging due to inconsistent terminology. In many cases, available entries could not be matched to the used classification of pathways and did therefore not enter the final dataset. Information on the habitat of each species is not directly linked to one location but to the taxon, and was retrieved from the original datasets when available. *Habitat* information was standardized according to the terms proposed in the SInAS workflow due to the absence of specific habitat terms from Darwin Core and categorized into *terrestrial*, *freshwater*, *marine* and *brackish* habitats.

The translation table for location names includes the standardized location names, their corresponding sub-divisions (i.e. states, provinces, departments, etc.) and alternative names including different spellings for each location and sub-division (Fig. 1). The inclusion of the sub-divisions allows for the up-scaling of records mapped at finer scales as provided for plants^49^ and ants^50^. The translation table also provides information of coarser spatial units, such as the WGSRPD and our own regional aggregation (Fig. 1). This setup allows flexibility, as regions can be adjusted by the user by modifying the translation table to accommodate any desired custom or published aggregation tailored to specific needs.

Taxon names were matched against the GBIF Backbone Taxonomy^26^, which is based on 105 sources, mostly nomenclatural authorities and taxonomic datasets and is primarily based on the Catalogue of Life Checklist. The provided taxon names were first compared to the GBIF Backbone Taxonomy to find exact matches. If no exact match was found, synonyms were checked and finally, if no matching synonym could be found, a fuzzy matching with high confidence was performed. If the matching was successful, the accepted taxon name and the respective taxonomic information (i.e. *species*, *genus*, *family*, *order*, *class*, *phylum* and *kingdom*) were retrieved and stored. Taxon names identified as synonyms were replaced with GBIF’s accepted taxon name and flagged as such. Through GBIF’s fuzzy matching, a *confidence* metric is calculated by comparing the provided taxon name with GBIF’s Backbone Taxonomy. Taxon names with a high level of *confidence* (i.e. confidence level of 100) were accepted, thus avoiding mismatches due to minor spelling errors. Homonyms (i.e. identical taxon names used for different taxa) were addressed by checking further information provided by the original datasets, such as the author name, the kingdom or the taxonomic group of the taxon if provided in the original source. Identified homonyms and those, which could not be resolved, are flagged as such in the final output. All taxonomic names that could not be matched were kept in the dataset. Original taxon names were kept in a separate column.

Finally, we grouped all taxa based on the taxonomic information retrieved from GBIF for each accepted taxon name. These taxon groups represent groups of species commonly used in biogeographical analyses, such as mammals, vascular plants or insects (*taxaGroup* in Table 2). Note that not all groups are monophyletic (i.e. descending from a single common ancestor) and instead may represent ecological groups, such as in the case of algae.

The *eventDate* informs about the first documented occurrence of a species in a location outside its native range. Entries provided as a single year were retained in their original format. Records provided as a time range with two dates were standardized by calculating the arithmetic mean of the two dates. Dates in other formats were standardized according to the guidelines used by Dyer *et al*.^11^, translating them into a single year.

In each standardization step, unresolved and non-matched terms were exported for reference and to allow cross-checking the corresponding records manually.

In the last step, the standardized datasets were merged into a single dataset. The merging process is based on the taxonomic names, locations and the corresponding establishment mean for each record. Records for a species in the same location were separated if they have different means of establishment, for example, if a species is reported as both *native* and *introduced* in the same location. However, we concatenated records categorized as *introduced* and *uncertain* as “*introduced; uncertain*”, thus keeping all available information about the alien status of a species in a single record. Additional details specific to the alien status of a species in a location, such as the degree of establishment and pathway, were assigned to the entries flagged as *introduced*. Entries with the same information on the taxonomic name, location and establishment mean were merged to avoid duplicates. When multiple *eventDates* were given for the same species in a location, we kept the earliest reported record.

Each record is attributed to its data source using the source’s abbreviated name (*datasetName*, detailed in Table 3) and its formal citation (*bibliographicCitation*). This citation refers to the data source, not the first publication for the individual record. To trace a specific record to its first publication, users should consult the data source listed under *datasetName*.

For more detailed information on the standardization of the data see Seebens *et al*.^19^.

## Data Records

The final dataset is SInAS 3.1.1. It represents a major extension of the existing SInAS dataset, which does not include native distributions and which has not been published as a scientific data paper with a proper description yet. The SInAS 3.1.1 dataset is stored on Zenodo^20^ (10.5281/zenodo.5562891) and consists of the following files:

The main data file is a single comma-separated file (.csv format), entitled “SInAS_3.1.1.csv”. The column names and entries are shown in Table 1. Each row represents a record for a taxon (alien or native) in a location.

A second file is composed in a single comma-separated file (.csv format), entitled “SInAS_3.1.1_FullTaxaList.csv”, The column names and entries are shown in Table 2. Each row corresponds to a retrieved taxon from a source dataset (Table 3).

The files “SInAS_3.1.1.csv” and “SInAS_3.1.1_FullTaxaList.csv” include the column *taxon*, which contains the scientific name of a taxon without authority and date after being matched and standardized (if available) against GBIF Backbone Taxonomy. If a taxon was not available for standardization in the GBIF Backbone Taxonomy, the column *taxon* retains the original name as provided by the source dataset. This column should be used as the primary reference for taxon names in our dataset.

The file “SInAS_3.1.1_FullTaxaList.csv” includes an entry for each taxon provided by a source dataset. If a taxon was successfully matched against the GBIF Backbone Taxonomy, we included the column *scientificName*, which contains the full scientific name of a taxon with authority and date information when available. Additional taxonomic information is included for these taxa. In cases where a taxon was not matched to the GBIF Backbone Taxonomy we were unable to retrieve its corresponding taxonomic information or assign it to a taxon group. These taxa are mostly varieties, crosses, subspecies or species complexes. Furthermore, we noticed that many of these taxa are ornamental plant crosses and variations, as well as parasitic microorganisms.

Each taxon and location has its unique identifier, which is consistent across files. To merge “SInAS_3.1.1_FullTaxaList.csv” and “AllLocations.gpkg” with the main output file “SInAS_3.1.1.csv”, the columns *taxonID* and *taxon*, and *locationID* and *location* should be merged, respectively.

### Data overview

The final dataset contains standardized information across 289 locations for 41,289 alien taxa. From these, we collected information on the alien distribution of 39,700 taxa and the native distribution of 21,345 taxa.

Altogether, there are currently 427,956 records in the dataset. From these, 174,034 (40.6%) records are flagged as introduced, 240,855 (56.2%) records as native, 8,183 (1.9%) as introduced and uncertain and 4,879 (1.1%) as uncertain. Additionally, 39,492 (9.2%) have information on the degree of establishment, 314,974 (73.6%) on the occurrence status, 2,558 (0.5%) on the pathway and 82,817 (19.3%) on the year of introduction. Furthermore, information on the habitat is available for 22,729 taxa (55.1% of total included taxa) in the dataset, with the possibility of multiple habitats being assigned to the same taxon.

**Fig. 3:**
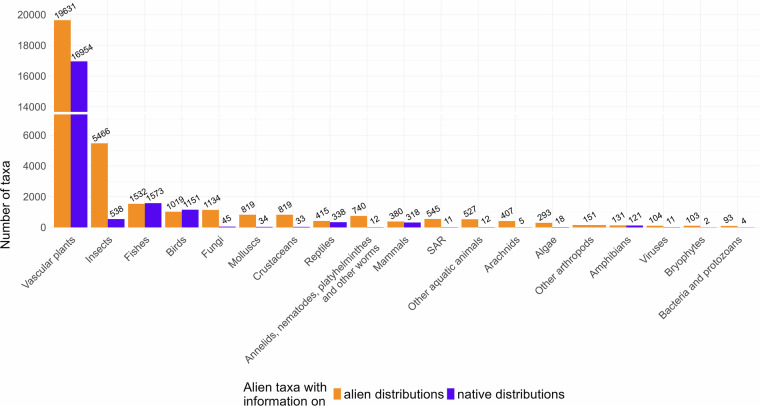
Number of taxa with information on alien or native distributions by taxa group included in this dataset. The counts represent the number of taxa matched with GBIF Backbone Taxonomy and assigned to a taxa group. Taxa that could not be matched are not present in this summary.

From the 41,289 taxa included in the dataset, 37,991 (92%) were matched and harmonized using the GBIF Backbone Taxonomy. The majority of taxa with either alien or native distributions information (Fig. 3) are vascular plants, arthropods and vertebrates. This pattern aligns with general trends in biodiversity and invasion biology research, where these taxonomic groups are generally better represented^51,52^.

In some cases, we retrieved information on native and alien distributions of a species but were not able to align the alien distribution to our spatial categorization. Therefore, some species only have native distributions in our dataset (n = 1,278).

**Fig. 4:**
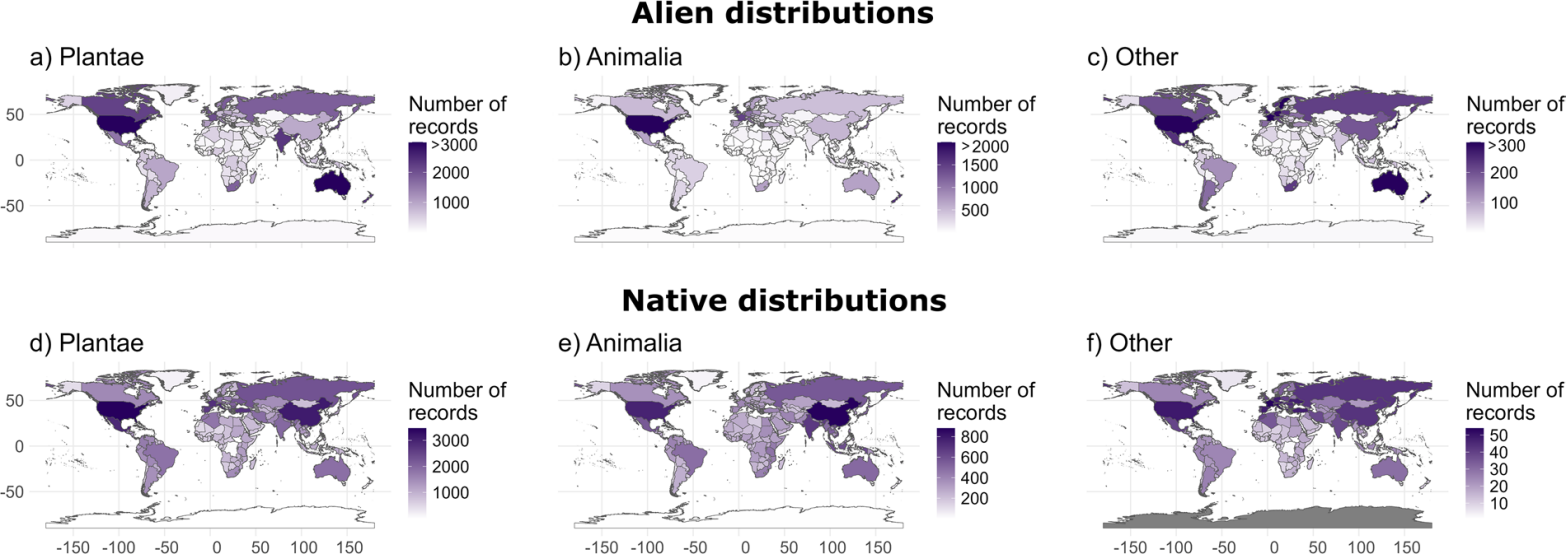
(**a**–**c**) Alien and (**d**–**f**) native distributions: Number of records (an alien taxon in a location) per region of introduction or origin. Locations without information are colored in grey. Note that the scales differ among each map. The category “Other” includes records for the kingdoms Fungi, Chromista, Bacteria and Protozoa, and for Viruses.

Distributional patterns differ among the native and alien distributions (Fig. 4). While records of alien distributions are predominantly concentrated in Northern America, Oceania and Europe, records of native distributions are more evenly distributed across geographies. For instance, many reported alien species originate from biodiversity-rich countries, such as those in Southern and Central America, Southern Asia and Sub-Saharan Africa.

## Technical Validation

We carefully evaluated available information on alien and native distributions and selected only maintained and curated data sources. In addition, only information about species distributions, invasion status and taxonomic information reported by expert-reviewed sources, such as peer-reviewed journals and IUCN Red List Assessments, were accessed and used to draw the information for this dataset. We prioritized the most recent, global-scaled and comprehensive sources to obtain the best up-to-date data.

To assure consistency and accuracy across the dataset, we implemented an established harmonization process for the taxonomic and geographical information, as well as for the characteristics regarding the alien status of a taxon in a location. This guarantees full transparency and reproducibility as well as uniformity across the dataset to ensure that the data adheres to consistent biodiversity standards.

## Usage Notes

### Workflow to create SInAS 3.1.1

The workflow to create the dataset is implemented in R and the scripts are provided on GitHub (see *Code availability*). Replicating the workflow as provided in GitHub requires opening the RStudio project file SInAS_workflow and running the file R/runWorkflow.R. A more detailed description is provided in a manual, which is provided with the code (see below).

In addition to R-scripts, the GitHub folder contains all required configuration files providing information about the final set of terms and locations. These are called translation tables and refer to the variables for *occurrenceStatus*, *establishmentMeans*, *degreeOfEstablishment, pathway* and *habitat*, and the list of *location* names, their variations and different sets of regions of aggregation. These files can be modified by the user to adjust the process of harmonization when re-running the workflow.

Finally, the GitHub folder contains a manual describing the structure of the files, as well as detailed information on how to execute the workflow and each of its steps.

The input files provided in GitHub are for demonstration purposes only and allow users to create a sample subset of the dataset. The full versions of all input files needed to replicate SInAS 3.1.1, as well as the corresponding configuration file for these input files, are available in the Zenodo repository of the dataset^20^. To reproduce SInAS 3.1.1, the input files need to be copied into the subfolder ‘Inputfiles’, while the configuration file needs to be copied into the subfolder ‘Config’.

### Limitations

The SInAS 3.1.1 dataset draws from a wide array of expert-validated sources. Still, users should be aware of inherent limitations relating to data availability and accuracy. Some datasets may not be regularly updated, leading to potential discrepancies in the current distribution or invasion status of alien species. Users are encouraged to consider the context of each dataset and verify key records against current primary data sources available.

Additionally, data completeness and quality may vary by taxonomic group and region due to regional differences in research infrastructure and monitoring efforts. Users accessing this dataset should consider these factors when using the data, and we explicitly welcome contributions that may enhance the dataset’s overall completeness, accuracy and reliability.

It is also important to note that the actual ranges of species are likely smaller and independent of the administrative borders (i.e. countries or sub-nations) used for this dataset. Consequently, the provided information overestimates the actual species ranges, which must be taken into account when assessing their distributions. Range maps of alien species distributions, such as those provided by GAVIA, DAMA and IUCN, can be accessed at the respective original sources for more fine-scaled distributions and used to calculate the proportion of range maps overlapping country polygons. This measure could offer a more nuanced view of species distributions within regions.

Some species distributions were extracted directly from range maps (i.e. IUCN and BirdLife). These are provided as comparatively coarse range maps. Mapping those to our spatial classification may lead to erroneous assignments of countries to the range of a species, particularly towards the borders of the ranges. Therefore, assignments of countries towards the borders of species ranges should be treated carefully.

## Data Availability

The SInAS 3.1.1 dataset is stored on Zenodo^20^ (10.5281/zenodo.5562891). The repository contains the main output files “SInAS_3.1.1.csv” and “SInAS_3.1.1_FullTaxaList.csv” (see *Data Records*), as well as lists of unresolved terms, taxa and locations in the zip file “All_Output_SInAS_3.1.1.zip”. The zip file “All_Inputfiles_SInAS_3.1.1.zip” include all input datasets^14,30–38,41,45,49,53^ and “All_Config_SInAS_3.1.1.zip” contains the configuration file to recreate SInAS 3.1.1 (see *Usage Notes: Workflow to create SInAS 3.1.1*). In addition, the repository provides a geopackage file with the polygons of the regions used in the SInAS dataset and a manual outlining the application of the workflow in detail.

## Code availability

The files needed to run the harmonization based on the SInAS workflow are stored on GitHub (https://github.com/hseebens/SInAS), which is linked to a Zenodo repository to obtain a DOI for each code release (10.5281/zenodo.3763221).
